# Identification of novel predictive factors for post surgical corneal haze

**DOI:** 10.1038/s41598-019-53123-3

**Published:** 2019-11-18

**Authors:** Nimisha R. Kumar, Pooja Khamar, Rohit Shetty, Ankit Sharma, Naren Shetty, Natasha Pahuja, Valsala Gopalakrishnan Abilash, Vishal Jhanji, Anuprita Ghosh, Rajiv R. Mohan, Rajani Kanth Vangala, Arkasubhra Ghosh

**Affiliations:** 1 GROW Research Laboratory, Narayana Nethralaya Foundation, Bangalore, India; 2 Cornea and Refractive Surgery Division, Narayana Nethralaya, Bangalore, India; 3 Department of Biomedical Sciences, School of Bio Sciences and Technology, VIT, Vellore, India; 4 Thrombosis Research Institute, Bangalore, India; 5 Singapore Eye Research Institute, Singapore, Singapore; 6 Department of Veterinary Medicine and Surgery, University of Missouri, Columbia, MO 65211 USA; 7 Mason Eye Institute, School of Medicine, University of Missouri, Columbia, MO 65212 USA; 8 Harry S Truman Veterans’ Memorial Hospital, Columbia, MO 65201 USA; 9 Department of Ophthalmology & Visual Sciences, The Chinese University of Hong Kong, Hong Kong, China; 10 Department of Ophthalmology, University of Pittsburgh School of Medicine, Pittsburgh, PA USA

**Keywords:** Corneal diseases, Biomarkers

## Abstract

Molecular factors altered in corneas that develop haze post refractive surgery have been described, but pre-existing factors that predispose clinically normal corneas to aberrant fibrosis post surgery and the role of the corneal epithelium remains unknown. We analyzed the global gene expression in epithelium collected intraoperatively from subjects undergoing photorefractive keratectomy. Subjects were grouped into those that developed haze 12 months post surgery (n = 6 eyes; haze predisposed) and those that did not develop haze in a similar follow up duration (n = 11 eyes; controls). Ontological analysis of 1100 upregulated and 1780 downregulated genes in the haze predisposed group revealed alterations in pathways associated with inflammation, *wnt* signaling, oxidative stress, nerve functions and extra cellular matrix remodeling. Novel factors such as PREX1, WNT3A, SOX17, GABRA1and PXDN were found to be significantly altered in haze predisposed subjects and those with active haze(n = 3), indicating their pro-fibrotic role. PREX1 was significantly upregulated in haze predisposed subjects. Ectopic expression of PREX1 in cultured human corneal epithelial cells enhanced their rate of wound healing while its ablation using shRNA reduced healing compared to matched controls. Recombinant TGFβ treatment in PREX1 overexpressing corneal cells led to enhanced αSMA expression and Vimentin phosphorylation while the converse was true for shPREX1 expressing cells. Our data identify a few novel factors in the corneal epithelium that may define a patient’s risk to developing post refractive corneal haze.

## Introduction

Corneal haze is clouding of the corneal layer post any infection or surgical insult affecting the quality of vision. Refractive vision correcting surgeries such as photorefractive keratectomy (PRK) and laser assisted *in situ* keratomileusis (LASIK) are performed on millions of eyes annually. Corneal haze is an unwanted adverse outcome with an incidence of 1.44%^1^. The nature and location of corneal haze is also associated with the type of preceding surgical procedure – corneal collagen crosslinking (CXL) is linked to mid-stromal haze, whereas PRK results in sub-epithelial haze^2^. Despite significant evidence available regarding the characteristics of corneal haze, the etiopathogenesis and predisposing factors are poorly understood in humans. *In vitro* and *in vivo* studies conducted to understand corneal haze post PRK or chemical burns have focused on primarily the modulation of the TGFβ^3^ pathway, inflammation^4^ and the extracellular matrix remodeling^5^. Clinical risk factors associated with post-refractive corneal haze includes high refractive error, higher ablation depth, smaller ablation zone^6^ and UV B exposure^7^. Administration of topical steroids is one of the most common prophylactic and post-operative therapeutic strategies to prevent and manage haze development. However, studies have shown that use of these drugs has not been efficacious^8^. Mitomycin C (MMC) has also been used after excimer ablation to manage haze with reasonable success but the safety of this drug with reference to the cytotoxic effects on stromal keratocytes and corneal endothelium remains a concern^9,10^.

The wound healing response is usually tightly controlled by various members of the TGF superfamily^11^ which are in turn regulated by growth factors such as PDGF, EGF, HGF, KGF etc^12^. Once wounded, the corneal stromal keratocytes undergo differentiation to myofibroblasts which perform repair functions^13^ such as collagen deposition and ECM remodeling. The corneal epithelium has been shown to significantly contribute to the wound healing process and development of myofibroblasts in the stroma by secreting cytokines and growth factors including TGFβ^14^. Proliferation and migration of stromal keratocytes to the wound site is mediated by factors secreted by the corneal epithelium^15^. Thus, if the corneal epithelium secretes unbalanced levels of regulatory factors, it may contribute to abnormal fibrotic response in the stromal cells by driving excessive myofibroblast formation, aberrant collagen deposition and extracellular matrix remodelling^16^. Hence, corneal epithelium could serve as repository of factors that may predispose clinically normal subjects undergoing refractive surgery to develop haze.

Previous studies in human samples have focused on analyzing the fibrotic corneas that underwent transplants^17,18^. However, these tissues represent the end stage of the fibrotic process^19^. Hence there is a distinct lack of prior knowledge regarding molecular and tissue factors that predispose clinically healthy human eyes to develop haze post refractive surgery. Animal models of corneal haze also adopt acute damage models such as 9D PRK^20^ and alkali burns^21^ (1 N NaOH) which precipitate an immediate, robust pro-fibrotic response, which precludes the study of pre-existing tissue specific factors that tilt the balance of the wound healing response in certain human corneas post insult. We therefore studied the altered status of pre-surgery gene expression in corneas of subjects undergoing refractive correction. The corneal epithelium from age, sex and duration of follow up matched subjects were obtained intra-operatively, prior to excimer laser ablation injury. Upon follow up, the subjects were grouped into those that developed haze and compared to those that did not by using microarray based gene expression analysis to identify novel factors that were altered in the haze predisposed group. It should be noted that this study in human subjects is only feasible in the corneal epithelium since it is debrided during the surgical procedure. This study reveals, for the first time, a set of factors whose pre-existing levels in the corneal tissue may possibly interact or influence known fibrotic mechanisms in corneal tissue post surgery leading to aberrant wound healing and haze.

## Results

### Clinical indices

**Figure 1:**
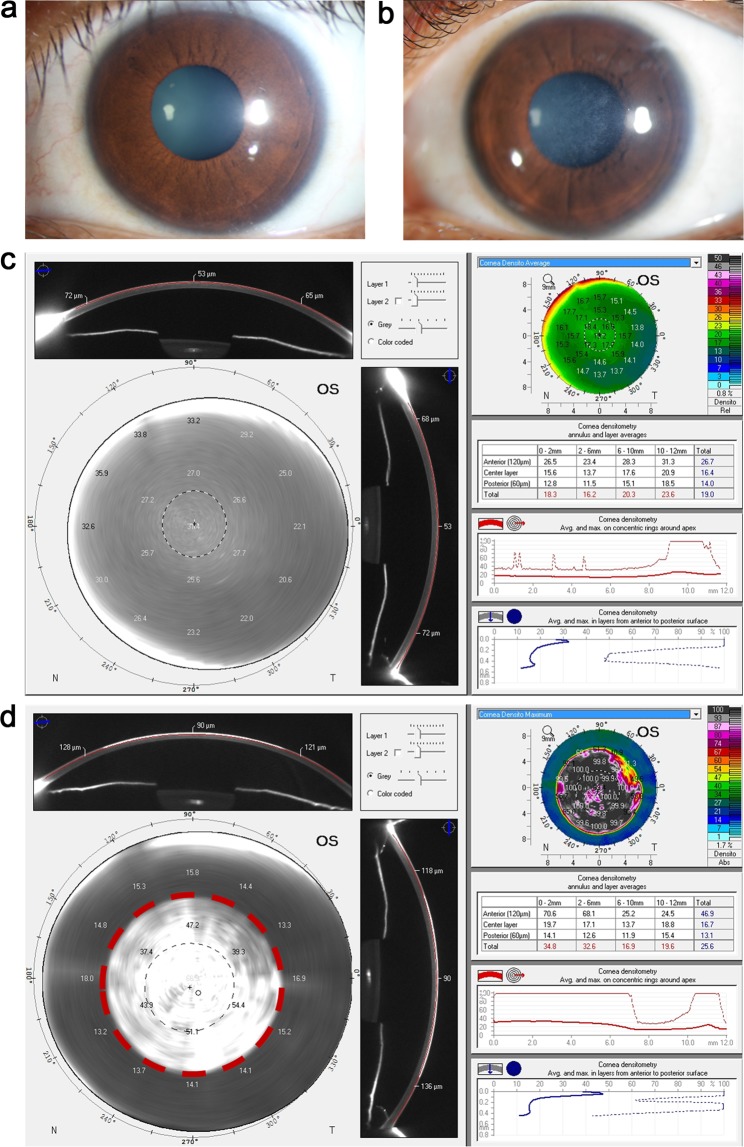
Clinical images illustrating corneas which underwent PRK surgery using Slit lamp bio microscopy (**a**) Control subject: clear cornea 12 months post PRK and (**b**) Corneal haze subject: cornea of grade 2 subepithelial corneal haze 12 months post PRK. To visualize post PRK haze, the densitometry mapping by Oculus Pentacam shows absolute values in different zones within normal range for control (**c**). Increase in Gray scale units (GY) at 0–2, 2–6- and 6–10-mm zone in the anterior 120 micrometer in corneal haze subject is shown (**d**). The area enclosed red dotted line demarcates haze. The corneal densitometry analysis gives the absolute values at different zones based on a colour scale. The corneal densitometry average values are represented in tabular and graphical format within the Pentacam readouts. The graph shows corresponding spikes to the increased grayscale values in different zones which represents the areas of increased back scatter caused by corneal haze (**d**) when compared to normal cornea (**c**).

The study comprised of 345 patients who underwent PRK between 2013 and 2018. Under this cohort of patients, eyes that underwent PRK for −2.00 D to −7.00 D of myopia were included. As per the guidelines, MMC was used for patients with ablation depth higher than 75 microns^22^. All subjects were screened during follow up for the development of vision compromising corneal haze. The demographic, refractive and keratometric parameters are listed in Table 1. The ‘haze predisposed’ group consisted of six eyes of three patients who had undergone PRK surgery and developed corneal haze during postoperative follow-up, and persisted for more than 6 months. Age and gender matched subjects who underwent the same procedure, but did not develop haze were considered as controls. The representative slit lamp bio-microscopy revealed grade 2 sub-epithelial haze along with 1.75 fold increase in gray scale units (GY) at 0–2, 2–6 and 6–10 mm zone in the anterior 120 micrometer Fig. 1(b–d) compared to gender, age and post-surgery duration control subject (n = 11) Fig. 1(a–c) seen at 6 months, post-operatively (representative image from indicated groups). The demographic, clinical and refractive parameters are described in Table 1. The keratometric indices like, K1, K2, Km and Kmax were comparable amongst the groups prior to surgery.

**Table 1:** Haze patient cohort demographics.

Clinical observations	HAZE patient cohort			
	Control (n = 11)	Haze predisposed (n = 6)	Post PRK Haze (n = 3)	p value
Age	25.73 ± 1.03	26 ± 2.22	24.33 ± 2.33	0.85
K1 (D)	44.14 ± 0.46	44.48 ± 0.45	43.63 ± 2.27	0.81
K2 (D)	45.24 ± 0.5	45.67 ± 0.63	45.27 ± 2.38	0.92
Km (D)	44.66 ± 0.47	45.05 ± 0.54	44.43 ± 2.32	0.89
K-Max (D)	45.65 ± 0.52	46.25 ± 0.59	46.43 ± 1.62	0.71
MRSE (D)	−3.34 ± 0.53	−4.42 ± 0.67	−6.5 ± 0.13	0.06
TCT (µm)	512.18 ± 5.78	502.17 ± 6.07	462.33 ± 28.18	0.02
BAD D	1.39 ± 0.16	1.61 ± 0.21	2.16 ± 0.67	0.21

The table provides mean ± sem values of the corneal thickness and corneal deformation assessed using Corvis-ST. K1, K2: Independent readings of corneal curvature by keratometry; Km and K-Max: Mean keratometry value and maximum keratometry value respectively. MRSE: Manifest refraction spherical equivalent, TCT: Thinnest Corneal Thickness; BAD D index is a measure of severity of keratoconus. The ANOVA p value column shows group statistics; pair wise statistics explained in the results section.

### Microarray and bioinformatic analysis

**Figure 2:**
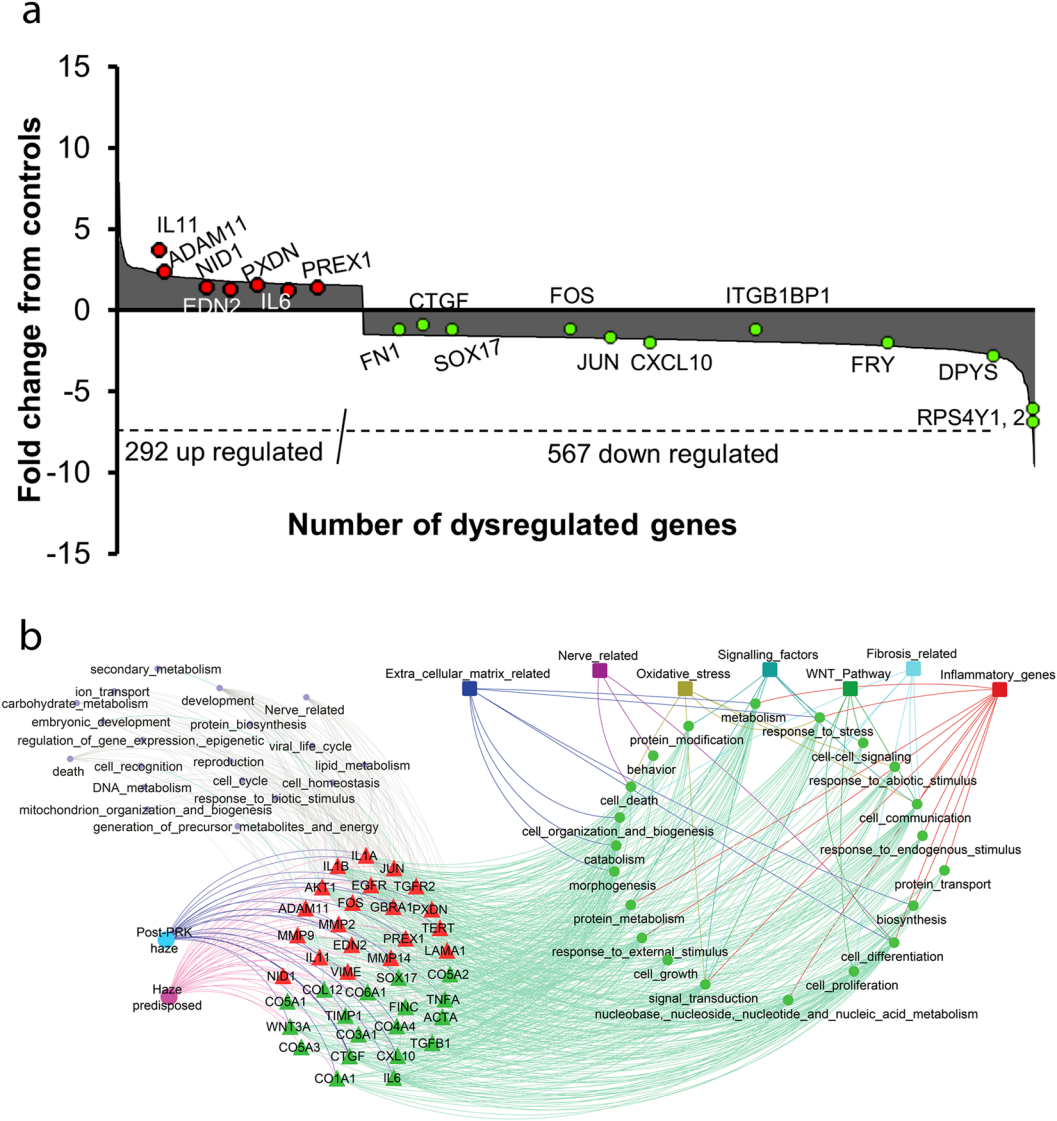
Microarray of the pooled mRNA samples from haze predisposed and control groups reveals 292 upregulated and 567 downregulated genes cut off >  ±2 fold change (p-value < 0.05). (**a**) Graph represents data of 859 genes differentially regulated. (**b**) STRING analysis of gene networks and pathways differentially regulated in haze predisposed and post PRK haze groups of patients. 38 differentially expressed genes (triangle shapes, red color signifies up regulation and green down regulation respectively) related to 21 molecular functions (green circles) were observed to be associated with 7 important pathways (square box).

The epithelium from the 3 ‘haze predisposed’ and 11 controls were used for microarray analysis. The microarray revealed 1100 up regulated and 1780 down regulated genes from the pooled sets of control and pre-disposed patient epithelia. Selection of differentially expressed genes with a cut off >  ±2 fold change (p-value < 0.05) resulted in 292 upregulated and 567 downregulated genes (Fig. 2a). These selected genes were used for network construction, of which 327 genes were found to have interactions in Protein-Protein Interaction (STRING) database (Supplementary fig. 1). In order to simplify and analyze the potential interactions, a total of 38 genes of which 15 genes from microarray (supplementary table 1a) and 23 genes from the wound healing literature (supplementary table 1b) were analysed using Gene Ontology (GO) to identify the important pathways and molecular interactions. 21 parent ontologies were found to intersect with 7 pathways (327 genes) associated with haze predisposed combined with post-PRK haze representing cross talk among pathways **(**Fig. 2b). A tabular representation showing different molecular functions like extra cellular matrix, nerve, signaling factors, oxidative stress, WNT pathway, fibrosis and inflammation related with the respective number of genes involved in each of these functional pathways is shown in Table 2.

**Table 2:** Microarray data categorized according to functional pathways

Pathways	Parent gene ontology	No. of genes
Extra cellular matrix related	Response to stress	23
	Metabolism	33
	Morphogenesis	26
	Cell differentiation	9
	Catabolism	18
	Cell organization and biogenesis	31
	Biosynthesis	18
Nerve related	Cell death	15
	Cell differentiation	9
	Behavior	9
Signaling factors	Response to stress	23
	Protein modification	17
	Protein metabolism	21
	Metabolism	33
	Signal transduction	29
	Cell communication	29
	Cell-cell signaling	8
Oxidative stress	Response to stress	23
	Cell death	15
	Signal transduction	29
	Cell communication	29
	Response to abiotic stimulus	17
WNT pathway	Cell proliferation	19
	Cell differentiation	9
	Signal transduction	29
	Cell communication	29
	Cell-cell signaling	8
Fibrosis related	Cell proliferation	19
	Signal transduction	29
	Cell communication	29
	Cell growth	9
	Cell organization and biogenesis	31
	Response to endogenous stimulus	18
Inflammatory genes	Response to stress	23
	Protein metabolism	21
	Metabolism	33
	Cell differentiation	30
	Signal transduction	29
	Cell communication	29
	Protein transport	11
	Response to external stimulus	25
	Nucleobase, nucleoside, nucleotide and nucleic acid metabolism	16
	Response to endogenous stimulus	18
	Biosynthesis	18

Numbers of genes expressed in the microarray data are associated with parent gene ontologies and their differential fold change are listed in the above table, representing a diverse functional pathway.

### Validation of microarray with mRNA expression in patient cohort

**Figure 3:**
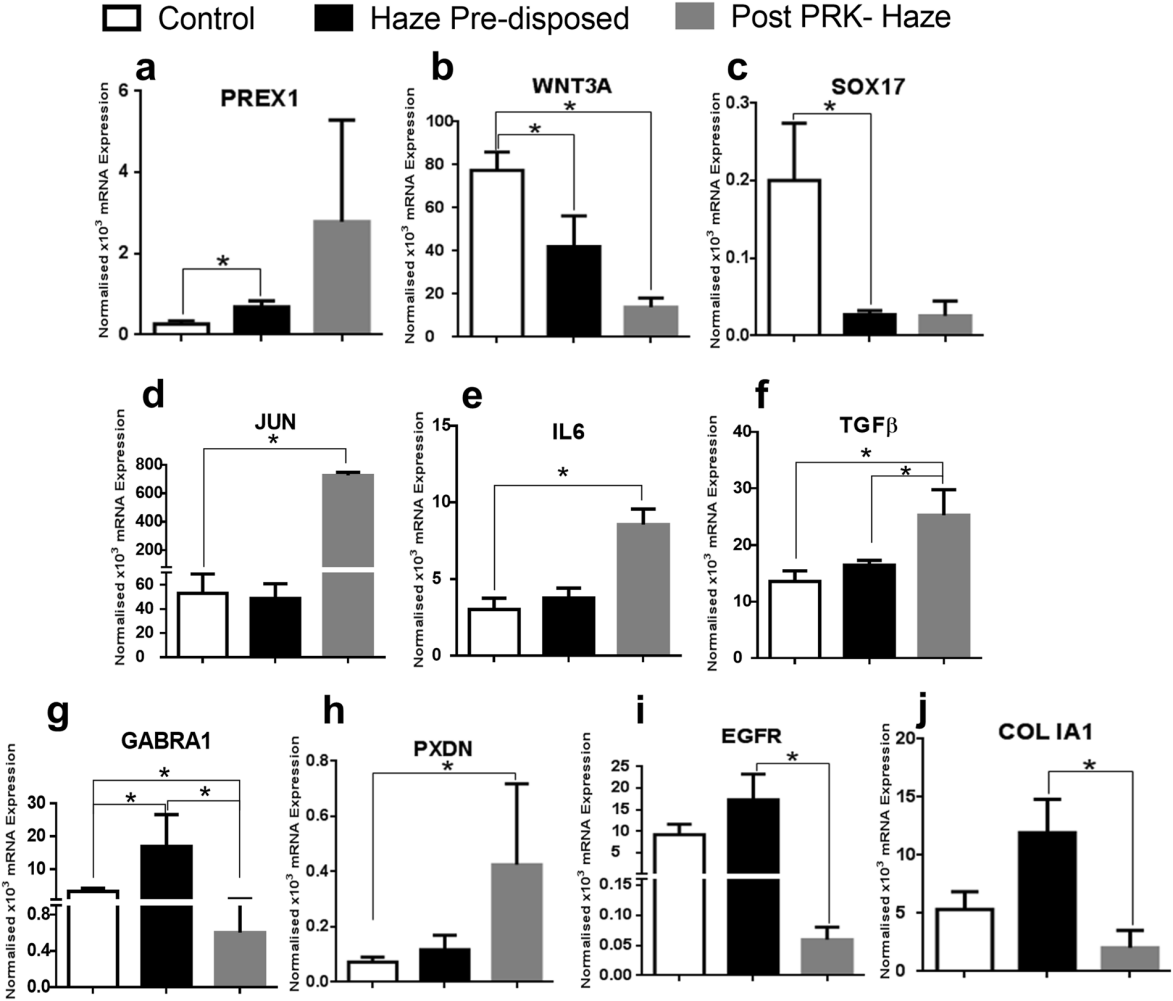
mRNA expression from corneal epithelium tissues collected intra-operatively. Differentially regulated transcript levels of (**a–j**) PREX1, WNT3A, SOX17, JUN, IL6, TGFβ, GABRA1, PXDN, EGFR and COLIA1 is represented. Bar graph consists of three groups namely, control (n = 11) represented by white bar, haze predisposed (n = 6) represented by black bar and post PRK haze (n = 3) subjects represented by grey bar. Data shown are mean ± SEM (*p < 0.05).

Transcription levels measured in corneal epithelium from normal PRK (controls, n = 11), haze predisposed (n = 6) and post PRK corneal haze (n = 3) patients (Fig. 3). Epithelium of patients who directly presented to the cornea clinic with vision compromising haze due to a prior PRK procedure were categorized as ‘post PRK haze’ group.

PREX1 (phosphatidylinositol-3,4,5-trisphosphate-dependent Rac exchange factor1) expression level was significantly (2.6 fold) higher in haze predisposed samples (mean ± SEM 0.68 ± 0.15, p = 0.03) as compared to controls (mean ± SEM 0.26 ± 0.08) which was further elevated to 10.7 fold in post PRK samples (mean ± SEM 2.79 ± 2.5) (Fig. 3a). Expression of WNT pathway members were significantly reduced across groups compared to controls. WNT3A (wingless type family member 3A) and its downstream target SOX17 (SRY (sex determining region Y)-box 17) were reduced by 0.5 and 0.1 fold in haze predisposed (mean ± SEM 42 ± 14.25, p = 0.03; 0.03 ± 0.01, p = 0.04) and 0.18, 0.12 folds in post PRK (mean ± SEM 13.94 ± 4.31, 0.03 ± 0.02) subjects respectively (Fig. 3b,c) compared to controls (mean ± SEM 77.49 ± 8.43; 0.2 ± 0.07). Proto- oncogene JUN (Fig. 3d), was 0.9 fold reduced in haze predisposed samples (mean ± SEM 48.73 ± 12.21) but significantly elevated to 13.7 folds in post PRK (mean ± SEM 725.42 ± 23.14, p = 0.04) compared to controls (mean ± SEM 52.91 ± 16.15). Interleukin 6(IL6), a classic inflammatory marker (Fig. 3e) expressed more across the groups being significantly 2.8 fold higher in post PRK (p = 0.03) and 1.3 fold in haze predisposed. Slight increase in TGFβ mRNA expression level (Fig. 3f) was observed; controls (mean ± SEM 13.6 ± 1.85) and haze predisposed group (mean ± SEM 16.45 ± 0.88) whereas a significant 1.86 fold increase in post PRK (mean ± SEM 25.26 ± 4.53, p = 0.03 compared with control and p = 0.04 compared with haze predisposed) indicated fibrosis. A unique neuronal gene GABRA1(gamma-aminobutyric acid (GABA) A receptor α1; Fig. 3g) was observed to be significant amongst the groups, i.e, from control (mean ± SEM 3.34 ± 0.94) increased to 5.1 fold in haze predisposed (16.94 ± 9.62, p = 0.03) and reduced to 0.18 fold in post PRK group (mean ± SEM 0.6 ± 0.44, p = 0.04 compared with control and p = 0.02 compared with haze predisposed). PXDN (peroxidasin), an adhesion molecule, revealed an increasing trend of 1.6 fold in haze predisposed (mean ± SEM 0.12 ± 0.05) and 5.9 fold in post PRK haze group (mean ± SEM 0.43 ± 0.29, p = 0.03) when compared to the controls (mean ± SEM 0.07 ± 0.02) (Fig. 3h). Extra cellular matrix remodeling factors like COL IA1(Collagen typeIα1) and EGFR (Epidermal growth factor receptor; Fig. 3i–j) showed an increased expression of 2.2 and 1.9fold in haze predisposed (mean ± SEM 11.92 ± 2.89, 17.3 ± 5.93) than controls (mean ± SEM 5.34 ± 1.52, 9.22 ± 2.41) and significantly reduced to 0.38 and 0.006 fold in post PRK group (mean ± SEM 2.02 ± 1.51, p = 0.03; 0.06 ± 0.02, p = 0.03) compared from haze predisposed. Based on the functional pathway, genes and their regulated fold change represented in mean ± sem values from controls, haze predisposed and post PRK group with their p values are listed in Table 3. Due to insufficient tissue available, validation of few indicated genes could be performed only in controls and haze predisposed groups (represented in Table 4 with p values).

**Table 3:** Table represents genes from microarray validated by mRNA expression (mean ± SEM) in control, haze predisposed and post PRK haze cohort and their respective p-values across the groups.

Functional pathways	Selected genes	Patient cohort gene expression (qPCR values)			p Values		
		Controls	Haze predisposed	Post PRK haze	Control vs haze predisposed	Control vs post PRK	Haze pre disposed vs Post PRK
Inflammatory related	IL11	0.23 ± 0.07	0.32 ± 0.2	0.15 ± 0.01	0.79	0.84	1
	IL1β	39.71 ± 1.79	37.71 ± 5.65	0.73 ± 0.03	0.64	0.48	0.08
	TNFα	0.33 ± 0.04	2.98 ± 1.68	0.23 ± 0.02	0.42	0.44	0.20
	CXCL10	1.24 ± 0.39	0.93 ± 0.35	0.48 ± 0.13	0.52	0.32	0.35
	TGFβR2	13.75 ± 1.92	15.65 ± 3.97	15 ± 0.71	0.87	0.60	0.64
	EDN2	1.54 ± 0.55	2.67 ± 2.22	0.22 ± 0.04	0.89	0.13	0.16
Nerve related	ADAM11	1.56 ± 0.34	3.26 ± 1.54	1.03 ± 0.05	0.20	0.67	0.06
	AKT1	0.04 ± 0.01	0.07 ± 0.02	0.05 ± 0.03	0.19	0.69	0.70
Fibrosis related	CTGF	2.49 ± 0.71	4.18 ± 2	50.64 ± 4.68	0.48	0.43	0.80
	ACTA2	55.6 ± 10.42	58.98 ± 10.72	31.47 ± 1.77	0.60	0.44	0.05
	BMP7	79.66 ± 4.89	82.91 ± 13.49	88.45 ± 4.59	0.83	0.74	1.00
	VIM	5.87 ± 0.7	8.8 ± 2.71	9.37 ± 0.61	0.46	0.07	0.70
	FN1	1.03 ± 0.32	1.84 ± 0.64	2.57 ± 0.27	0.25	0.12	0.44
Extra cellular matrix related	MMP14	196.03 ± 16.12	243.02 ± 37.93	305.3 ± 25.74	0.22	0.81	0.64
	TIMP1	0.12 ± 0.01	0.13 ± 0.02	0.07 ± 0.03	0.28	0.23	0.08
	MMP9	0.58 ± 0.11	0.91 ± 0.24	0.35 ± 0.32	0.20	0.74	0.32

**Table 4:** Validation of genes from microarray in control and haze predisposed cohort using real time quantitative PCR.

Functional pathways	Selected genes	Patient cohort gene expression (qPCR values)		
		Controls	Haze predisposed	p Values Control vs Haze predisposed
Signaling factor	FOS	0.5 ± 0.13	0.88 ± 0.1	0.23
Extra cellular matrix related	NID1	1.13 ± 0.25	2.73 ± 0.39	0.01
	LAMA1	1.28 ± 0.43	3.62 ± 0.75	0.02
	LAMA5	32.81 ± 7.14	23.18 ± 6.8	0.45
	COL3A1	6.55 ± 2.82	0.6 ± 0.2	0.09
	COL5A1	4.36 ± 1.3	2.92 ± 0.26	1.00
	COL5A2	20.91 ± 3.51	13.55 ± 2.89	0.19
	COL5A3	48.2 ± 10.28	44.18 ± 4.48	0.83
	COL6A1	3.31 ± 0.92	1.5 ± 0.84	0.19
	COL12	81.96 ± 15.34	40.27 ± 9.1	0.04

Table consists of gene names and the regulated fold change observed in the patient cohort consisting of control (normal PRK who does not develop haze) and haze predisposed (normal PRK who develop haze). Highlighted in bold p-value represents statistical significance.

### Over expression of PREX1 promotes cell migration while inhibition of PREX1 delays wound closure in human corneal epithelial cells

**Figure 4:**
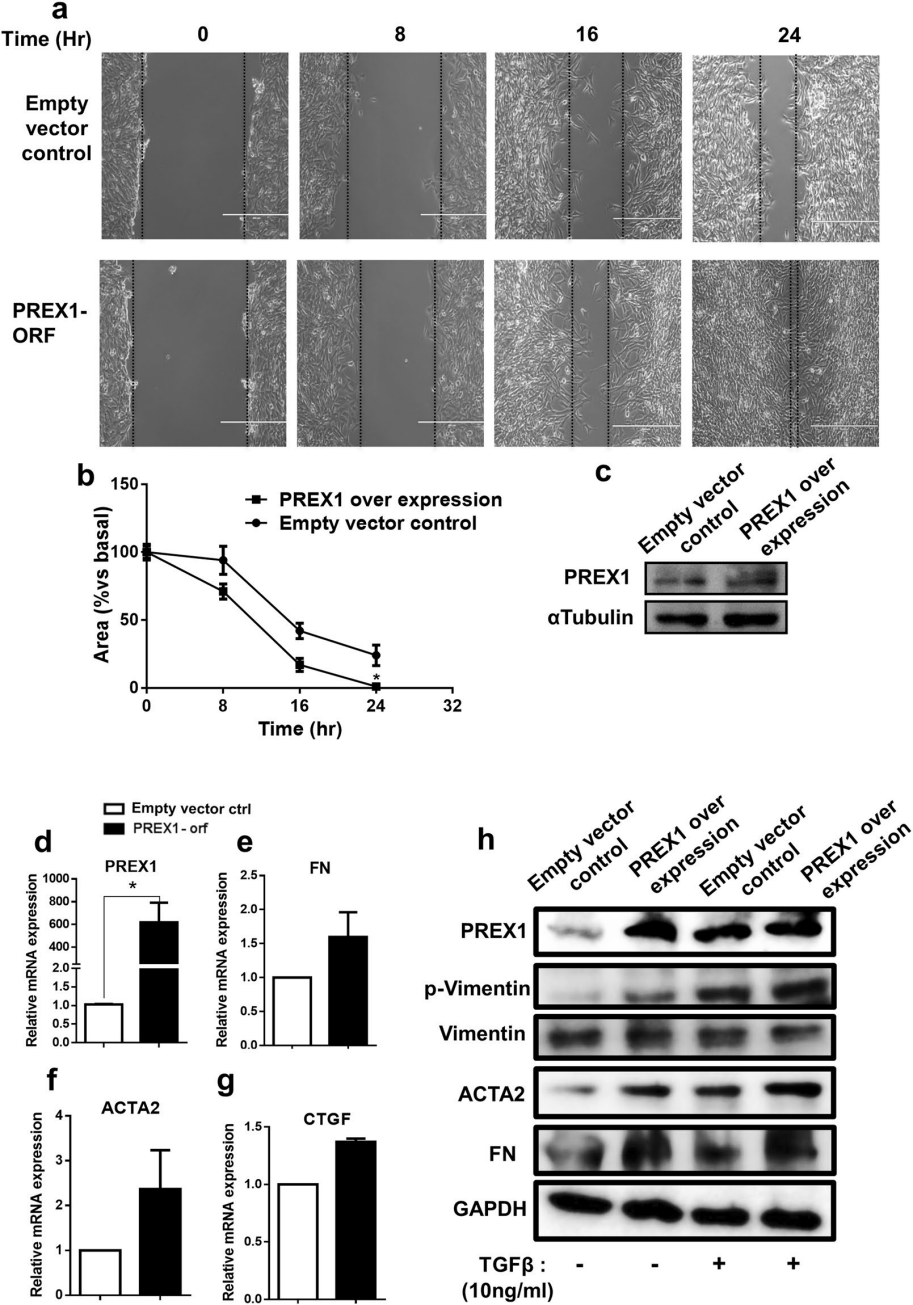
Over expression of PREX1 enhances migration of cultured human corneal epithelial cells (HCE). Lipofectamine mediated transfection was carried on HCE cells using PREX1 over expression (ORF) and empty plasmid served as control. A scratch wound assay was performed and (**a**) microphotographs were taken at 0, 8, 16 and 24 hr. (**b**) Analysis of wound closure area in empty control vs PREX1 ORF. (**c**) A representative western blot showing PREX1 protein and α-tubulin served as endogenous control (cropped from Supplementary figure 2a, lane 3 and 4 from blot 1&2; marked by white border). q-PCR analysis showing fold changes of (**d–g**) PREX1, FN, ACTA2 and CTGF. Data shown are mean ± SEM (*p < 0.05 ). (**h**) Effect of TGFβ on PREX1 overexpressing cultured human epithelial cells and empty plasmid control. Transfected cells were treated with and without TGFβ for 24 h. Representative western blot images showing protein levels of PREX1, p-Vimentin, total Vimentin, α-smooth muscle actin (ACTA2), fibronectin (FN) and GAPDH served as endogenous control.

The physiological relevance of PREX1 in human corneal epithelial cells was assessed using loss and gain of function in reference to wound healing. Ectopic expression of PREX1 in human corneal cells by transfection caused faster migration and healing of a scratch wound compared to control vector transfected cells when monitored at 0, 8, 16 and 24 h (Fig. 4a,b). PREX1 expression is shown to be significantly elevated in transfected cells compared to controls (p = 0.02) in HCE cells (Fig. 4c,d). Fibrotic markers were slightly elevated with fibronectin (FN) 1.6 fold, α-smooth muscle actin (ACTA2) 2.4 fold and connective tissue growth factor (CTGF) 1.37 fold in PREX1 expressing HCE cells (Fig. 4e–g). We observed increased fibrosis associated proteins ACTA2 and FN with elevated phosphorylation of vimentin in PREX1 overexpressing HCE (Fig. 4h). Furthermore, treatment of HCE cells overexpressing PREX1 with recombinant TGFβ for 24 hrs resulted in higher levels of such as ACTA2, FN and phosphorylated vimentin compared to controls (Fig. 4h).

**Figure 5:**
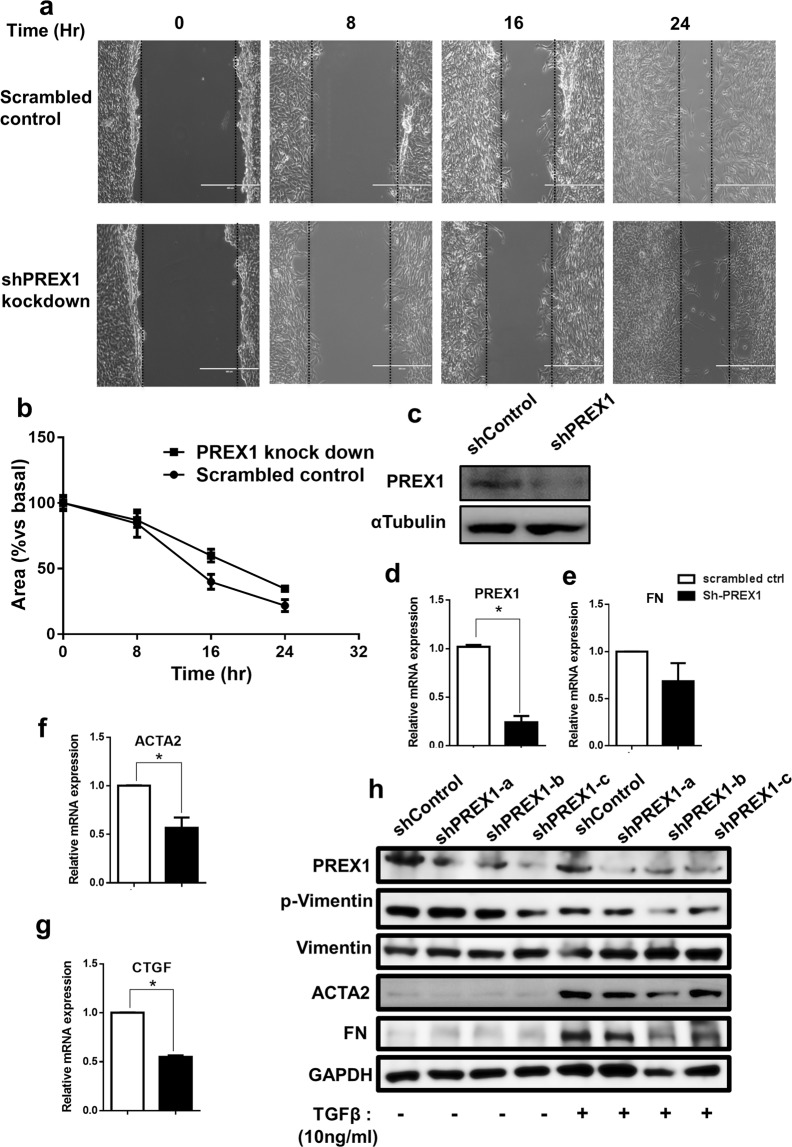
PREX1 ablation delays wound closure in cultured human corneal epithelial cells (HCE). Short hairpin (Sh) plasmids for PREX1 knock down and scrambled plasmid serving as control were used for transfection. (**a**) Microphotographs demonstrating wound status at 0, 8, 16 and 24 hr after scratching. (**b**) Analysis of wound closure area in scrambled control v/s Sh-PREX1 transfected cells. (**c**) Western blot illustrating PREX1 protein levels in scrambled control and sh-PREX1 transfected cells (cropped from Supplementary figure 2b, lane 6 and 7 from blot3&4, marked by white border). q-PCR showing fold changes of PREX1, FN, ACTA2 and CTGF (**d–g**). Data shown are mean ± SEM (*p < 0.05). (**h**) Role of TGFβ on PREX1 knockdown and scrambled plasmid control in HCE cells. Transfected cells were treated with and without TGFβ for 24 h. Immunoblot image showing protein levels of PREX1, fibronectin (FN), α-smooth muscle actin (ACTA2), p-Vimentin, total Vimentin and GAPDH served as endogenous control.

HCE cells transfected with PREX1 shRNA exhibited reduced cell migration and wound healing compared to scrambled shRNA control transfected cells, when monitored over 24 hours (Fig. 5a,b). ShRNA mediated ablation reduced levels of PREX1 expression to 0.24 folds (Fig. 5c,d). PREX1 ablated cells also had reduced expression of fibrotic genes, like FN 0.68 fold, ACTA2 0.56 fold (p = 0.01) and CTGF 0.54 fold (p < 0.0001) (Fig. 5e–g) compared to controls. Reduced phosphorylation of vimentin was observed upon recombinant TGFβ treatment in PREX1 shRNA HCE cells compared to matched control cells (Fig. 5h). Further, reduced ACTA2 & FN protein levels were also observed in PREX1 knockdown cells. The TGFβ treatment induced increase in ACTA2 & FN protein was slightly reduced levels in shRNA PREX1 cells compared to matched controls (Fig. 5h).

## Discussion

The corneal epithelial cells are the most metabolically active cells of the cornea which can respond to a variety of stimuli, stress and injury. They are also responsible for secreting the factors such as IL1α, PDGF, etc. which are required for activating the stromal keratocytes to initiate a wound healing response^23^. Animal models have focused primarily on the modulation of TGFβ pathway^24^, inflammation and the extracellular matrix during the healing process after acute injury such as high diopter refractive surgery^20^ or deep chemical burns^21^. It is generally thought that post injury; key factors like IL1β, TGFβ, and PDGF drive the deposition of excessive collagen and proteoglycans that result in haze or scar formation^25^. Numerous animal^26–28^ and human studies^29–31^ have illustrated the risk factors and ultra-structural observations after development of corneal haze. Refractive surgery is commonly practiced around the world for vision correction. While all pre-operative care is taken to ensure that only healthy eyes undergo refractive surgeries such as PRK, a small proportion of patients still develop vision compromising corneal haze. Thus, there is a lack of information regarding why some clinically healthy corneas undergoing surgery develop aberrant post surgical wound healing. We hypothesized that this aberrant corneal healing may be due to pre-existing alterations in the molecular profile of such subjects. This altered molecular profile may aid our understanding of alternative pathways that drive the development of corneal haze, which could help predict the outcome of surgeries.

Further, in the context of corneal wound healing and development of haze, more experimental information is available regarding the stromal cell activation^5^ as compared to the epithelium. Gene expression from the epithelium has been critical for understanding molecular mechanisms in diseases like inflammatory bowel disease, air way diseases, breast and ovarian cancer^32–34^. Moreover, epithelial-stromal cellular interactions have been reported in corneal wound healing response^35^. In rodent models, the role of altered epithelial control of pro-fibrotic pathways has been shown in corneal wound healing^36^. We therefore tested, if the pre-existing molecular profile of epithelial cells can affect the development of post surgical corneal haze in healthy human subjects undergoing PRK matched for age, surgical ablation and duration of follow up. Corneal haze following stromal ablation is associated with primarily the TGF-β signaling pathway which contains numerous ligands, receptors and signaling molecules^37^. We performed global transcriptome analysis of the patient epithelia that developed haze within 12 months of observation post surgery and compared them with controls who had clear vision. Our data shows that in subjects that went on to develop haze, a unique set of genes belonging to various signaling pathways were altered. Some of these alterations were further exaggerated in samples obtained after haze has developed, indicating their functional role in aberrant wound healing. We found reduced expression (0.75 fold in the haze predisposed group, 0.39 fold in post PRK group) of CXCL10 (chemokine (C-X-C motif) ligand 10), an IFNɣ responsive gene which participates in T_H_17 signaling indicating that innate immune responses were not heightened in these subjects. CXCL10 contributes to innate defense of the cornea against *P. aeruginosa* infection^38^, hence the lack of its activation precludes infections or aberrant inflammatory immune response in the haze pathogenesis. Certain inflammatory genes like IL11(Interleukin 11), TNFα (Tumor necrosis factor), pro-inflammatory peptide EDN2 (Endothelin 2) were slightly elevated (1.41, 1.17 and 1.73 fold) in haze predisposed patient tissues. IL11 was shown to be upregulated in retinal and corneal cells upon TNFα + IL1 induction^39^. EDN2 is reported to contribute in cystic fibrosis^40^ but its role in the cornea has not been explored. CTGF is known to promote corneal scar formation^41^ and we observed a 20 fold increased expression in post PRK haze epithelial tissues compared to controls or haze predisposed group. The lack of significant alteration in well known pro-fibrotic or inflammatory genes in the haze predisposed samples agrees with their clinically observed healthy status at the time of surgery. However, we found a set of other, novel genes that were altered in these haze predisposed subjects.

GABA receptors were identified in chick corneas^42^ and we also observed a significant expression of GABRA1 in corneal epithelial tissues, yet its purpose is an unexplored question. Proto oncogenes like JUN (p = 0.04) and FOS (1.8 fold) increased expressions are observed in haze predisposed epithelia. Immunolocalization of c-Fos and c-Jun protein in transcriptionally activated epithelial cells occurs during healing of corneal wounds and epithelial defects in Wistar rats^43^. Our data suggests that genes like WNT3A and SOX17, involved in Wnt/β-catenin signaling pathway, are significantly compromised in pre-disposed and post PRK haze groups. Expression and effects of Wnt/β-catenin signaling are based on tissue type^44^ which cross-talk with TGFβ signaling^45^ observed in oligodendrocyte progenitor cells, MLE-15 cells and SV40 immortalized mouse lung epithelial cell^46^. As reported by Corada *et al*.^47^ and Chew *et al*.^48^, SOX17 is a complex transcription factor which acts upstream of Notch system and suppresses cyclin D1 expression and proliferation downstream of the canonical Wnt signaling. PXDN, an adhesion molecule which is significantly increased in haze predisposed patient epithelia is involved in ECM formation and reported to promote tumor growth^49^. Peterfi *et al*. has reported ECM modification due to PXDN secretion by myofibroblasts for wound repair and tissue fibrosis in a murine model of kidney fibrosis^50^.

Our results show that PREX1, which encodes Dbl (diffuse B-cell lymphoma) family of Rho guanine exchange factors (RhoGEF), can be a predisposing factor for developing corneal haze. It is significantly increased in haze predisposed samples and is further increased in post PRK haze tissue. PREX1 is a mediator of TGFβ−1 signaling, acting as a signaling factor involved in both inflammatory and fibrogenic pathways of pulmonary fibrosis^51^. PREX1 can lead to invasiveness and metastasis of the tumor cells, reported in luminal subtype breast cancer^52^, glioblastoma cells^53^, melanoma tumor tissue and murine model^54^. Since most of these novel genes we found did not have documented function in human corneal healing, we chose to study the effect of PREX1 modulation in an *in vitro* wound healing model. Our experimental data demonstrates that ectopic PREX1 expression caused an exaggerated wound healing response in HCE while shRNA mediated ablation of PREX1 under the same conditions caused a delay in healing and migration. Notably, we observed that PREX1 elevation caused fold increase in ACTA2, FN and CTGF mRNA levels. Consequently, PREX1 overexpression caused increased ACTA2 and FN protein levels with concomitant increase in phosphorylated Vimentin. On the other hand, loss of PREX1 caused a significant reduction in ACTA2(p = 0.01) and CTGF (p < 0.001) while FN showed a similar trend. TGFβ can induce expression of CTGF^55^ and ACTA2^56^ during corneal wound healing. CTGF works as an upstream mediator of FN and ACTA2 and collectively enhances the attachment and migration of corneal epithelial cells^57^. FN can independently control TGFβ mediated pro-fibrotic signaling by controlling ECM formation and deposits of LTBPs and TGFβ^58^. We found TGFβ treatment to induce PREX1 in HCE cells. Further, when cells overexpressing PREX1 were treated with TGFβ, we found expression of FN and ACTA2 to be further enhanced with increase in phosphorylated vimentin. Thus, it is plausible that pre-existing higher levels of PREX1 can cause enhanced FN, ACTA2 and CTGF induction leading to a potent pro-fibrotic response post surgery.

In conclusion, our data reveals a set of novel molecular factors in cornea associated with corneal haze after refractive surgery. The network analysis of these genes suggests a number of new and complex interactions hitherto unknown in corneal wound biology which may have relevance towards clinical applications in the future. Importantly, one of these newly discovered factors, PREX1, is shown to regulate wound healing and also expression of fibrosis associated genes, linking it with TGFβ signaling in the human cornea. We note however, that the number of human samples used in this study are small and the results should be confirmed further. Therefore, future studies validating these observations in a larger, independent patient cohort and relevant animal models are warranted.

## Methods

All patient samples were collected after obtaining an informed, written consent from participants, prior approval of the institutional ethics committee and adhered to guidelines of the Declaration of Helsinki. The study cohort was selected from patients who reported to the cornea clinic at Narayana Nethralaya, Bangalore, India.

### Study cohort inclusion and exclusion criteria

Patients greater than 18 years of age with stable refraction for the past 12 months were included. Patient with clinical signs of keratoconus, dry eye, abnormal corneal topography, presence of epithelium damage, history of previous ocular surgery or corneal or anterior segment pathologies, history of ocular infection, keloids, connective tissue disorder or hypertrophic scars, ocular surface allergy within 6 months prior to surgery were excluded. Those subjects on chronic systemic corticosteroids or immunosuppressive therapy were also excluded.

### Surgical procedure and postoperative follow up

All patients who met the patient selection criteria underwent standard PRK procedure by a single surgeon (RS). The eyelids were retracted with a lid speculum and the eyelashes were everted using sterile surgical eye drape. Topical anesthetic eye drops 0.5% proparacaine hydrochloride (Paracaine, Sunways, India Pvt. Ltd) was instilled. The epithelium over central 9 mm of cornea was debrided using a mechanical scraper. The epithelium thus removed was collected in a sterile microfuge tube in balanced salt solution (BSS) and stored at −80 °C. Thereafter the refractive correction was performed using 193 nm excimer laser with Wavelight EX500 (Alcon Laboratories, Fort Worth, TX). A bandage soft contact lens (BCL; Ciba Vision, Duluth, GA) was applied for 3 to 5 days after surgery. Topical moxifloxacin hydrochloride 0.5% eye drops (Vigamox, Alcon, Bayer Pharma AG) and topical fluorometholone 0.1% (FML, Allergan, Inc) were prescribed. FML was tapered gradually over 12 weeks. Postoperative follow-up was scheduled at 5 days, 2 weeks, 1 month, 3 months, 6 months, 1 year and every year thereafter. Stromal haze was clinically assessed subjectively based on the haze grading system reported by Fantes *et al*.^59^: grade 0, completely clear cornea; grade 0.5, trace haze, seen with careful oblique illumination with slit- lamp biomicroscopy; grade 1, more prominent haze, not interfering with visibility of fine iris details; grade 2, mild obscuration of iris details; grade 3, moderate obscuration of the iris and lens; and grade 4, completely opaque stroma in the area of ablation. Uncorrected visual acuity and best-corrected visual acuity (BCVA) was measured using a Snellen acuity chart and represented in LogMar equivalent for analysis. Subjects presenting with corneal haze underwent Phototherapeutic keratectomy (PTK) or Femto assisted lamellar keratectomy (FALK) subsequently.

### Patient epithelium study groups

The epithelium collected during surgery from patients who developed corneal haze within 12 months after undergoing PRK were categorized as ‘haze predisposed’. Epithelium from age, gender and duration post treatment matched patients who continued to maintain corneal transparency were chosen as controls. Epithelium of patients who directly presented to the cornea clinic with vision compromising haze due to a prior PRK procedure were categorized as ‘post PRK haze’ group.

### Microarray and bioinformatics

Total RNA was isolated from the collected samples using Qiagen’s RNeasy minikit (Cat#74104). The RNA quality (QA/QC) was checked using bioanalyzer tape station and RNA integrity number was determined. Agilent ‘one-color microarray-based gene expression’ protocol using human 8 × 60 K array slides were used for gene expression analysis. cRNA purification was done using Qiagen’s RNeasy minikit (Cat#74104). Random hexamer method of labeling (Agilent’s Quick-Amp labeling Kit (p/n5190–0442)) was done followed by T7 promoter based-linear amplification to generate labeled complementary RNA. Hybridzation was done using Agilent’s *in situ* hybridzation kit 5188–5242 followed by Agilent GE wash buffer 1 and 2. The slides were scanned and feature extraction performed in Agilent Microarray scanner. The extracted data was analyzed using GeneSpring GX 12.6.1 Software. In each sample the log transformed intensity values for each probe were subtracted by the calculated 75th percentile value of the respective array and expression values were obtained. Genes with at least 2 fold change and p-value < 0.05 were considered for constructing the gene-gene network interactions that were identified using STRING database. The network was constructed using Cytoscape 3.2.1 (Supplementary figure 1).

The network was constructed by retrieving the UNIPROT data files of each of the 38 genes (Table 3). The gene ontologies (GO terms) and parent ontologies of each gene was extracted using CateGOrizer and were matched to GO terms associated with 7 different pathways. A network was constructed by creating an input file of interactions between gene – parent ontology and parent ontology – pathway using Cytoscape 3.2.1 (Fig. 2b).

### Human corneal epithelium cell culture

Human corneal epithelium cell (HCE) line was a kind gift from Dr. Rajiv Mohan, University of Missouri (Columbia, Missouri, USA). Cells were cultured under standard conditions using a 1:1 mixture of Dulbecco’s modified Eagle medium (DMEM) and Nutrient Mixture F12 (Gibco, Grand Island, NY) supplemented with 10% fetal bovine serum (FBS, certified USA Origin), 0.3 mg/ml L-glutamine (Gibco, Invitrogen), 0.1 mg/ml streptomycin, and 1000 IU/ml penicillin (Gibco, Invitrogen).

### Wound healing assay and transfection

HCE cells were plated in six well plates and transfected with 3 µg of either control (empty, scrambled) or PREX1 overexpression and PREX1 shRNA (transOMIC technologies inc., AL, USA) using Lipofectamine –LTX PLUS reagent (Invitrogen, CA, USA) according to manufacturer’s protocol and grown to confluence. A linear scratch was made on the cells using a sterile 20 μL pipette tip^60^ replenished with DMEM/F12 and microphotographs (EVOS™ FL, Invitrogen™, CA,USA**)** were taken at 0, 8, 16 and 24 hr after scratch. Cell migration across the wound was calculated by measuring reduction of wound area compared to the start of the experiment (0 hr). All the experiments were done in triplicates and the data represented as means ± SEM. Further, transfected cells were treated with recombinant TGFβ (10 ng/ml) for 24Hr and subjected to immunoblot analysis.

### Isolation of RNA, cDNA synthesis and real-time PCR

HCE cells were washed twice with PBS; thereafter, 1 ml trypsin was added directly to each well. Detached cells were collected and centrifuged for 1.5 minutes. The isolation of mRNA and analysis of gene expressions follows. Briefly, total RNA was extracted from cells and patient epithelia using TRIZOL method according to the manufacturer’s instructions (Invitrogen, Carlsbad, CA), followed by quantification and quality assessment. In addition, cDNA was synthesized using Superscript III (Life Technologies, Carlsbad, CA) cDNA conversion kit. The quantitative real-time PCR cycle includes pre-incubation at 95 °C for 3 minutes, followed by 40 amplification cycles at 95 °C for 10 seconds, annealing and extension at 58 °C for 30 sec with dissociation curve analysis at 65 °C to 95 °C increment 0.5 °C for 0.05 minutes using a CFX ConnectTM real-time PCR detection system (Bio-Rad, Philadelphia, PA) using SYBR Green Assay (KAPA SYBR Fast qPCR master mix: KAPA Biosystems, Wilmington, MA). Human β-actin was used as housekeeping reference gene.

### Western blot

HCE cells transfected with PREX1 over expression and shRNA along with vector controls were washed with PBS and lysed using 80 µl of radio-immunoprecipitation (RIPA) assay buffer (G-Biosciences, USA) containing protease and phosphatase inhibitors (Roche life science, USA). 40 µg of protein were run on SDS PAGE, transferred onto PVDF membrane blocked with 5% non-fat dry milk powder in PBST followed by overnight incubation with antibodies against PREX1 (1:500, Abcam#124231), α-Tubulin (1:1000, Cell Signaling #3873), α-smooth muscle Actin (1:500, Abcam #7817), phospho vimentin (1:1000, Cell signaling #12569), Vimentin (1:1000, santa cruz #32322),Fibronectin (1:1000, Abcam #2413), GAPDH (1:3000, Abcam #9485). The secondary antibodies (anti-rabbit, anti-mouse) were used to visualize the band (ImageQuant LAS 500, GE Healthcare Life Sciences, USA).

### Statistical analysis

All statistical analysis was performed with MedCalc v12.5.0 (MedCalc, Inc., Ostend, Belgium) and graphs were made using GraphPad Prizm 6.0 (GraphPad Software, Inc., La Jolla, CA, USA). As the patient cohort size was small, Mann Whitney (independent) test was run for analysis. ImageJ was used to calculate wound closure area.

### Ethics approval and consent to participate

Written informed consent was obtained from all the study participants. The study was approved by the Narayana Nethralaya Institutional Review Board and was performed as per institutional ethics guidelines and in accordance with the tenets of the Declaration of Helsinki.

## Supplementary information

Supplementary table 1

Supplementary dataset 1

## Data Availability

Microarray data to share it for research use shall be made available upon request.
