# Supplementary material for: Identification of novel predictive factors for post surgical corneal haze
Source: Sci Rep. 2019 Nov 18;9:16980. doi: 10.1038/s41598-019-53123-3 (PMC6861263; doi:10.1038/s41598-019-53123-3)
Supplement: Supplementary file 1 — Supplementary table 1 [file 41598_2019_53123_MOESM1_ESM.docx]

**Identification of novel predictive factors for post surgical corneal haze**

Nimisha R. Kumar^1,3^, Pooja Khamar^2^, Rohit Shetty^2,5^, Ankit Sharma^4^, Naren Shetty^2^, Natasha Pahuja^2^, Valsala Gopalakrishnan Abilash^3^, Vishal Jhanji^9^, Anuprita Ghosh^1^, Rajiv R Mohan^6,7,8^, Rajani Kanth Vangala^4^ and Arkasubhra Ghosh^1,5,^*

^1^GROW Research Laboratory, Narayana Nethralaya Foundation, Bangalore, India

^2^Cornea and Refractive Surgery Division, Narayana Nethralaya, Bangalore, India

^3^Department of Biomedical Sciences, School of Bio Sciences and Technology, VIT, Vellore, India

^4^Thrombosis Research Institute, Bangalore, India

^5^Singapore Eye Research Institute, Singapore

^6^Department of Veterinary Medicine and Surgery, University of Missouri, Columbia, MO, 65211 USA

^7^Mason Eye Institute, School of Medicine, University of Missouri, Columbia, MO 65212 USA

^8^Harry S Truman Veterans’ Memorial Hospital, Columbia, MO 65201

^9^Department of Ophthalmology & Visual Sciences, The Chinese University of Hong Kong, Hong Kong, China; Department of Ophthalmology, University of Pittsburgh School of Medicine, Pittsburgh, PA, USA.

***Corresponding author:**

Arkasubhra Ghosh, MSc, PhD

GROW Research Laboratory

Narayana Nethralaya Foundation, Narayana Health City,

# 258/A, Bommasandra, Hosur Road, Bangalore - 560 099 - INDIA.

Email: [arkasubhra@narayananethralaya.com](mailto:arkasubhra@narayananethralaya.com)

**Disclosure:** The authors have no financial disclosures or conflicts for interest to declare.

**Supplementary table1 (a):**  Represents list of 15 genes selected from microarray with their gene names and respective fold change values based on cut off > ±2 fold change (p-value<0.05)

| **S.no** | **Selected genes** | **Gene name** | **Regulated fold change values from microarray** |
| --- | --- | --- | --- |
| 1 | IL11 | Interleukin 11 | 3.9287033 |
| 2 | ADAM11 | ADAM metallopeptidase domain 11 | 3.5146933 |
| 3 | NID1 | Nidogen 1 | 2.596683 |
| 4 | EDN2 | Endothelin 2 | 2.5694585 |
| 5 | PXDN | Peroxidasin | 2.394092 |
| 6 | PREX1 | Phosphatidylinositol-3,4,5-trisphosphate-dependent Rac exchange factor 1 | 1.806623 |
| 7 | GABRA1 | Gamma-aminobutyric acid (GABA) A receptor, alpha 1 (GABRA1), transcript variant 3 | 1.4887972 |
| 8 | LAMA1 | Laminin, alpha 1 | 1.1277332 |
| 9 | AKT1 | v-akt murine thymoma viral oncogene homolog 1 | 0.81685543 |
| 10 | TGFβR2 | Transforming growth factor, beta receptor II | -0.7197604 |
| 11 | CXCL10 | Chemokine (C-X-C motif) ligand 10 | -1.1612573 |
| 12 | SOX17 | SRY (sex determining region Y)-box 17 | -1.8566742 |
| 13 | CTGF | Connective tissue growth factor | -2.3748293 |
| 14 | FOS | FBJ murine osteosarcoma viral oncogene homolog | -2.6987405 |
| 15 | JUN | Jun proto-oncogene | -3.4296188 |

**Supplementary table1 (b):** Represents 23 gene names with their known functions in wound healing and respective fold changes in microarray

| **S.no** | **Selected genes** | **Gene name** | **Regulated fold change values from microarray** |
| --- | --- | --- | --- |
| 1 | IL6 | Interleukin 6 | 1.881801 |
| 2 | COL5A3 | Collagen, type V, alpha 3 | 1.121002 |
| 3 | WNT3A | Wnt family member 3A; wingless-type MMTV integration site family, member 3A | 0.654665 |
| 4 | COL4A1 | Collagen, type IV, alpha 1 | 0.566932 |
| 5 | ACTA2 | Actin, alpha 2, smooth muscle, aorta | 0.39981 |
| 6 | TNFα | Tumor necrosis factor | 0.372458 |
| 7 | EGFR | Epidermal growth factor receptor | 0.346153 |
| 8 | COL6A1 | Collagen, type VI, alpha 1 | 0.273212 |
| 9 | LAMA5 | Laminin, alpha 5 | 0.130382 |
| 10 | MMP14 | Matrix metallopeptidase 14 | 0.115628 |
| 11 | TIMP1 | Metallopeptidase inhibitor 1 | 0.020782 |
| 12 | BMP7 | Bone morphogenetic protein 7 | -0.00721 |
| 13 | COL5A2 | Collagen, type V, alpha 2 | -0.04 |
| 14 | COL5A1 | Collagen, type V, alpha 1 | -0.04648 |
| 15 | MMP9 | Matrix metallopeptidase 9 | -0.05938 |
| 16 | IL1B | Interleukin 1, beta | -0.11039 |
| 17 | COL1A1 | Collagen, type I, alpha 1 | -0.11044 |
| 18 | TGFβ | Transforming growth factor, beta 1 | -0.19911 |
| 19 | COL12 | Collagen, type XII, alpha 1 | -0.21725 |
| 20 | MMP2 | Matrix metallopeptidase 2 | -0.23683 |
| 21 | COL3A1 | Collagen, type III, alpha 1 | -0.25931 |
| 22 | VIM | Vimentin | -1.07423 |
| 23 | FN1 | Fibronectin 1 | -1.25012 |
