# Supplementary material for: Identification of novel predictive factors for post surgical corneal haze
Source: Sci Rep. 2019 Nov 18;9:16980. doi: 10.1038/s41598-019-53123-3 (PMC6861263; doi:10.1038/s41598-019-53123-3)
Supplement: Supplementary file 2 — Supplementary dataset 1 [file 41598_2019_53123_MOESM2_ESM.pdf]

Identification of novel predictive factors for post surgical corneal haze

Nimisha R. Kumar[1,3], Pooja Khamar[2], Rohit Shetty[2,5], Ankit Sharma[4], Naren Shetty[2], Natasha Pahuja[2], Valsala Gopalakrishnan Abilash[3], Vishal Jhanji[9], Anuprita Ghosh[1], Rajiv R Mohan[6,7,8], Rajani Kanth Vangala[4] and Arkasubhra Ghosh[1,5,*]

[1]GROW Research Laboratory, Narayana Nethralaya Foundation, Bangalore, India
[2]Cornea and Refractive Surgery Division, Narayana Nethralaya, Bangalore, India
[3]Department of Biomedical Sciences, School of Bio Sciences and Technology, VIT, Vellore, India
[4]Thrombosis Research Institute, Bangalore, India
[5]Singapore Eye Research Institute, Singapore
[6]Department of Veterinary Medicine and Surgery, University of Missouri, Columbia, MO, 65211 USA
[7]Mason Eye Institute, School of Medicine, University of Missouri, Columbia, MO 65212 USA
[8]Harry S Truman Veterans' Memorial Hospital, Columbia, MO 65201
[9]Department of Ophthalmology & Visual Sciences, The Chinese University of Hong Kong, Hong Kong, China; Department of Ophthalmology, University of Pittsburgh School of Medicine, Pittsburgh, PA, USA.

***Corresponding author:**
Arkasubhra Ghosh, MSc, PhD
GROW Research Laboratory
Narayana Nethralaya Foundation, Narayana Health City,
258/A, Bommasandra, Hosur Road, Bangalore - 560 099 - INDIA.
Email: arkasubhra@narayananethralaya.com

Disclosure: The authors have no financial disclosures or conflicts for interest to declare.

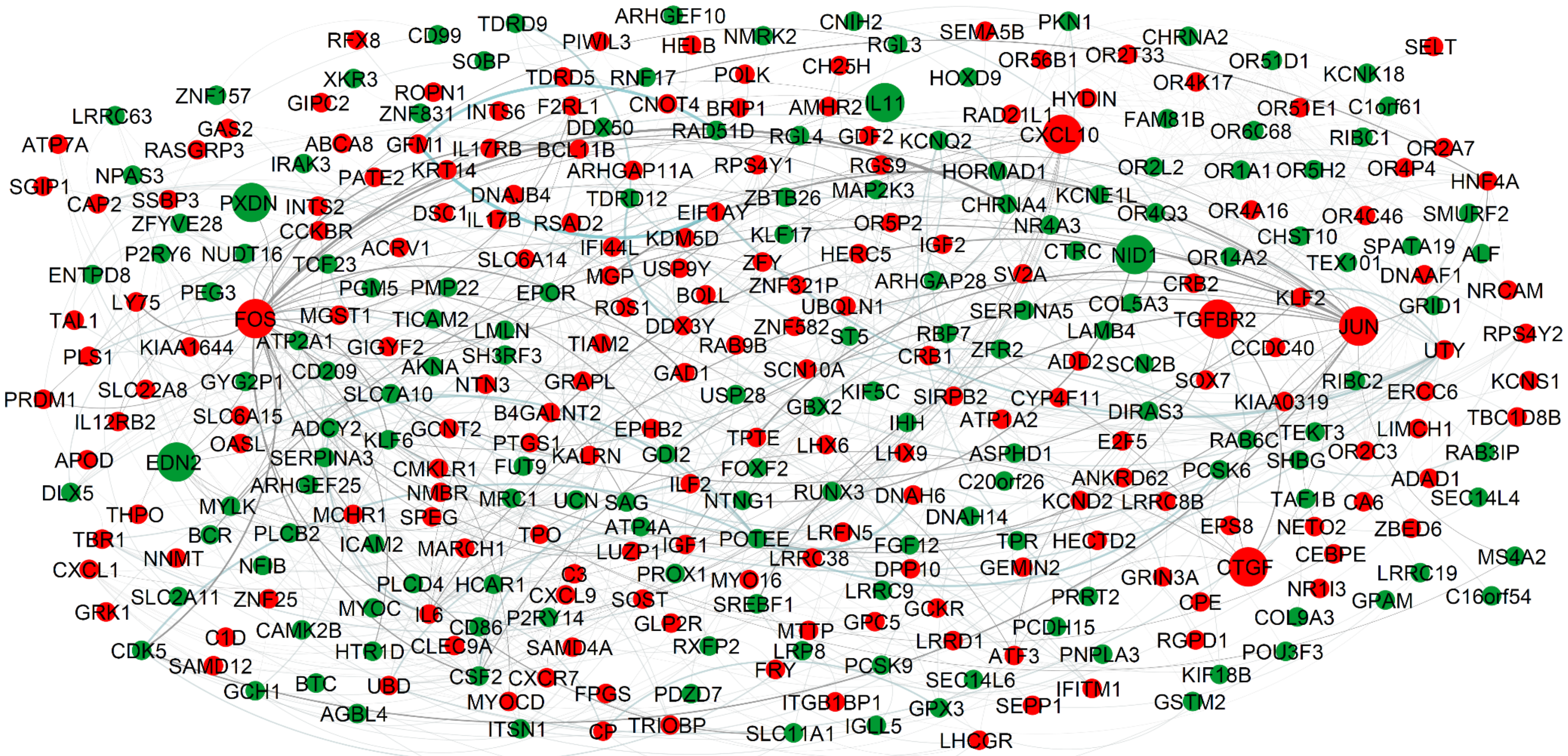

Supplementary figure1: Represents the network analysis showing 327 differentially expressed genes cut off > ±2 fold change (p-value<0.05) in microarray and their protein- protein interactions. The network analysis suggested that c-FOS, IL6, c-JUN, CXCL10, EDN2, IL1 and IL11 with node degrees of 34, 26, 23, 13, 9, 8 and 5 forms the backbone of the network architecture. The size of the circle (node) represents the fold change between control and haze predisposition (red color signifies down regulation and green color signifies up regulation).

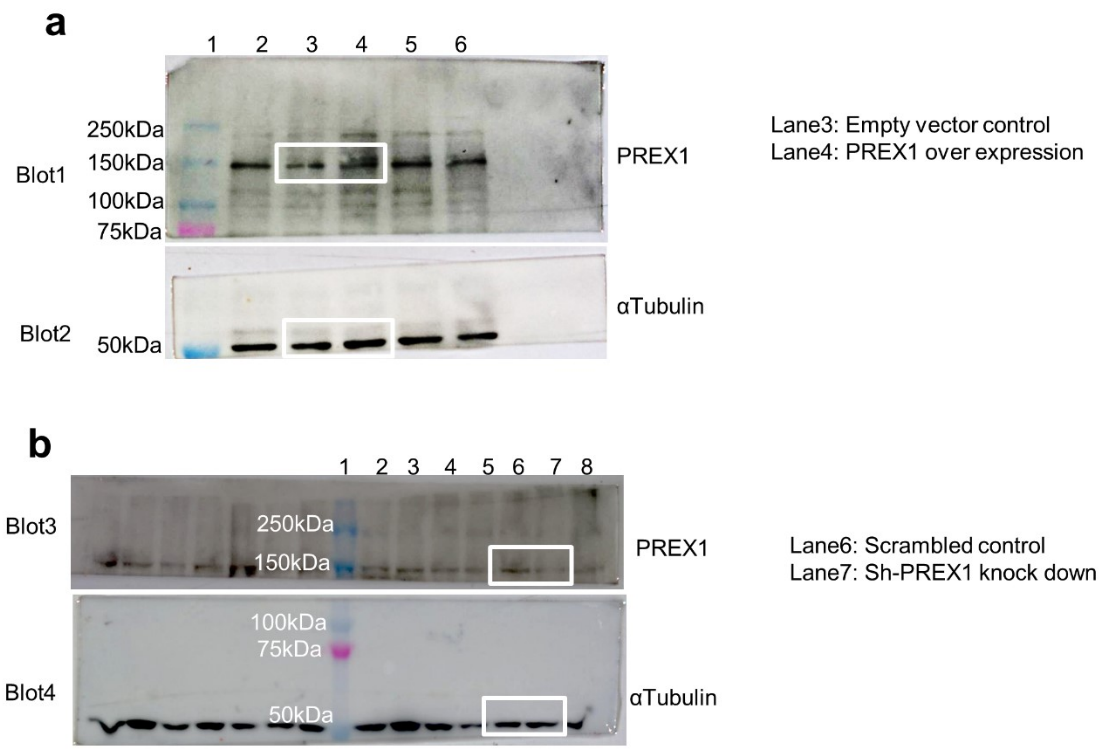

Supplementary figure2: Represents unedited western blot images used in the figure 4c and 5c. Panel (a) consists of blot1 representing PREX1 protein expression from empty vector transfected control (lane3) and PREX1 over expression plasmid transfected (lane4) and corresponding α-tubulin in blot2 marked within white square box, shown in figure4c. Panel (b) consists of blot3 representing PREX1 protein in scrambled control (lane 6) and sh-PREX1 knock down (lane 7) with their corresponding α-tubulin in blot4, delineated with white box and shown in figure5c.

c

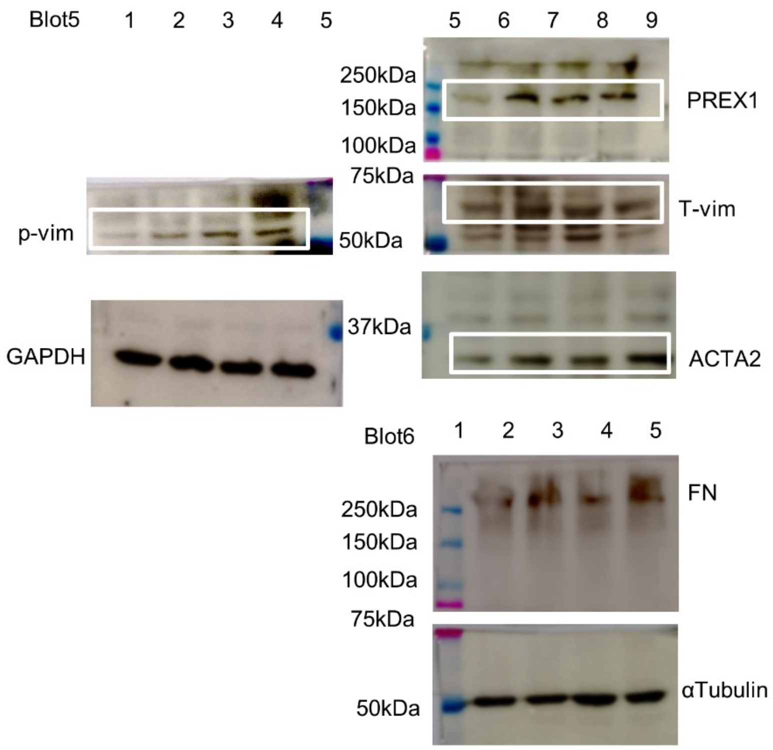

Lane1: Empty vector control
Lane2: PREX1 over expression
Lane3: Empty vector control +TGFβ
Lane4: PREX1 over expression+ TGFβ
Lane5: Ladder
Lane6: Empty vector control
Lane7: PREX1 over expression
Lane8: Empty vector control +TGFβ
Lane9: PREX1 over expression+ TGFβ

d

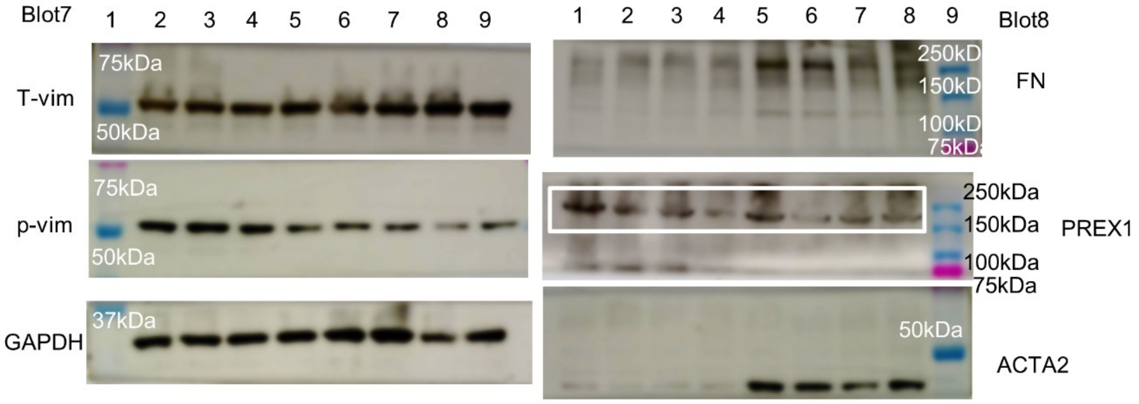

Lane1: Ladder
Lane2: Scrambled control
Lane3: Sh-PREX1 knock down
Lane4: : Sh-PREX1 knock down
Lane5: : Sh-PREX1 knock down
Lane6: Scrambled control + TGFβ
Lane7: Sh-PREX1 knock down +TGFβ
Lane8: Sh-PREX1 knock down +TGFβ
Lane9: Sh-PREX1 knock down +TGFβ

Lane1: Scrambled control
Lane2: Sh-PREX1 knock down
Lane3: : Sh-PREX1 knock down
Lane4: : Sh-PREX1 knock down
Lane5: Scrambled control + TGFβ
Lane6: Sh-PREX1 knock down +TGFβ
Lane7: Sh-PREX1 knock down +TGFβ
Lane8: Sh-PREX1 knock down +TGFβ
Lane9: Ladder

Supplementary figure2: Represents unedited western blot images used in the figure 4h and 5h. Panel (c) consists of blot5 representing PREX1 overexpression with empty controls in human corneal epithelial cells. Lane1-9 represents the sample conditions loaded for protein analysis of p-vim, GAPDH, FN, PREX1, T-vim and ACTA2 marked within white square box, shown in figure 4h. Blot6 represents FN and its corresponding loading control tubulin. Panel (d) consists of blot 7 representing PREX1 knockdowns with scrambled control in human corneal epithelial cells. Lane1-9 represents the sample conditions loaded for protein analysis of T-vim, p-vim and GAPDH. Blot 8, lane 1-9 represents the sample conditions loaded for protein analysis of FN, PREX1 and ACTA2 marked within white square box, shown in figure 5h.
